# Dual role of exosomes in neurodegenerative diseases: a molecular bridge between neuroinflammation and transmission of pathological proteins

**DOI:** 10.3389/fneur.2025.1708655

**Published:** 2026-01-14

**Authors:** Wei An, Ze Jin, Yan Li

**Affiliations:** 1 Heilongjiang University of Chinese Medicine, Harbin, Heilongjiang, China; 2 The Fifth Department of Acupuncture and Moxibustion, The Second Affiliated Hospital of Heilongjiang University of Chinese Medicine, Harbin, Heilongjiang, China; 3 Department of Otolaryngology, First Affiliated Hospital of Heilongjiang University of Chinese Medicine, Harbin, Heilongjiang, China

**Keywords:** exosome, extracellular vesicles, neurodegenerative diseases, neuroinflammation, pathological proteins

## Abstract

Neurodegenerative diseases (NDDs) are complex disorders characterized by the progressive loss of neuronal function. Their pathological mechanisms involve multiple levels, including neuroinflammation, abnormal protein aggregation, and disrupted cell signaling. Diseases such as Alzheimer’s disease (AD), Parkinson’s disease (PD), Amyotrophic Lateral Sclerosis (ALS), multiple sclerosis (MS), and prion diseases not only severely impact patients’ quality of life but also pose significant challenges for medical research due to their complex pathogenesis and the lack of effective treatments. In recent years, extracellular vesicles (EVs), particularly exosomes, have garnered increasing attention for their critical role in cell-to-cell communication. Exosomes are membrane-enclosed nanovesicles approximately 30–150 nm in diameter that can carry proteins, lipids, nucleic acids, and other bioactive molecules, influencing recipient cells through paracrine or distant signaling. This review aims to summarize the roles of exosomes as mediators of neuroinflammation and as vehicles for intercellular transmission of pathogenic proteins in neurodegenerative diseases.

## Introduction

Neurodegenerative diseases (NDDs) are a complex group of disorders primarily characterized by the progressive degeneration of neurons (1), encompassing various clinical subtypes such as Alzheimer’s Disease (AD), Parkinson’s Disease (PD), and Amyotrophic Lateral Sclerosis (ALS) (2, 3). Despite phenotypic differences, their pathological mechanisms revolve around two core axes (4): abnormal protein aggregation and the cascade activation of chronic neuroinflammation (5). The former constitutes the common molecular pathological basis of various NDDs (6–8). Notably, these two axes are not independent. Instead, they are interconnected through complex molecular interactions, forming positive feedback loops that jointly drive disease progression (9).

Abnormal protein conformation and aggregation are hallmark molecular features of NDDs (10). Classic examples include: extracellular plaque deposition of *β*-amyloid and hyperphosphorylated Tau forming neurofibrillary tangles in AD (11); accumulation of *α*-synuclein (α-Syn) forming Lewy bodies in PD (12); and cytoplasmic inclusions of Transactive Response DNA-binding Protein 43 (TDP-43) in ALS (12). These abnormal proteins damage neurons by disrupting organelle function, inducing oxidative stress, and impairing synaptic plasticity (1). More pathologically significant is the spatially specific transcellular spread of these proteins. For instance, Tau propagates along neuroanatomical pathways from the entorhinal cortex to the hippocampus and neocortex, which is consistent with Braak staging (13, 14); A-syn, on the other hand, exhibits a distinct progression: it spreads from the brainstem to the cortex, and its spatial distribution correlates strongly with the stages of clinical symptoms (15, 16). Evidence indicates that exosome-mediated transmembrane transport plays a crucial role in this spread, alongside synaptic connections (17); Neuron-derived exosomes can effectively encapsulate toxic proteins such as Aβ oligomers, phosphorylated Tau, or oligomerized *α*-syn. They enter recipient cells via membrane fusion or clathrin-mediated endocytosis, and can even cross the blood–brain barrier (BBB) into systemic circulation, thereby enabling the systemic dissemination of pathological factors (18).

Neuroinflammation exceeds the traditional view of a passive response in NDDs and is now recognized as a key amplifier of disease progression (19). Pathological activation of microglia and astrocytes creates a chronic inflammatory microenvironment, releasing pro-inflammatory cytokines, reactive oxygen species (ROS), and complement components, which not only exacerbate neuronal damage but also promote further accumulation of abnormal proteins (20, 21). Molecular studies show that Aβ oligomers activate microglial surface TLR4/CD36 complexes, triggering NLRP3 inflammasome assembly and leading to a burst of IL-1β release (22–24); similarly, *α*-syn binds to glial TLR2 receptors, activating the NF-κB pathway and inducing sustained synthesis of pro-inflammatory mediators (25, 26). This inflammation-protein aggregation feedback loop is significantly intensified during late stages of disease due to BBB disruption and infiltration of peripheral immune cells, ultimately resulting in irreversible neurostructural damage (27–29).

Exosomes exhibit a complex bidirectional regulatory role within NDDs’ pathological networks (30). As pathological carriers, they serve as “molecular messengers of pathogenesis,” transporting toxic proteins (e.g., A*β*42, phosphorylated Tau) for transcellular propagation, and mediating prion-like spreading of *α*-syn (16, 31). They act as endogenous regulatory systems by delivering anti-inflammatory factors (e.g., IL-10, TGF-β) or neurotrophic molecules (e.g., brain-derived neurotrophic factor, BDNF), suppressing inflammation and promoting neuronal repair (32). Of particular note, owing to their inherent ability to penetrate the BBB and their low immunogenicity, exosomes are highly valuable for diagnostics and therapeutics (33): exosomes in cerebrospinal fluid or peripheral blood can enrich disease-specific biomarkers, providing early detection windows. Studies report significant exosomal protein and RNA differences between PD patients and healthy individuals (34). For example, Sun and colleagues, through extensive targeted metabolomics analysis, identified marked differences in metabolites within serum and brain exosomes from MPTP-induced PD mice compared to healthy controls, with 69 differentially expressed metabolites in serum and 148 in brain tissue (34).

## Biological functions and secretion of exosomes

**Figure 1:**
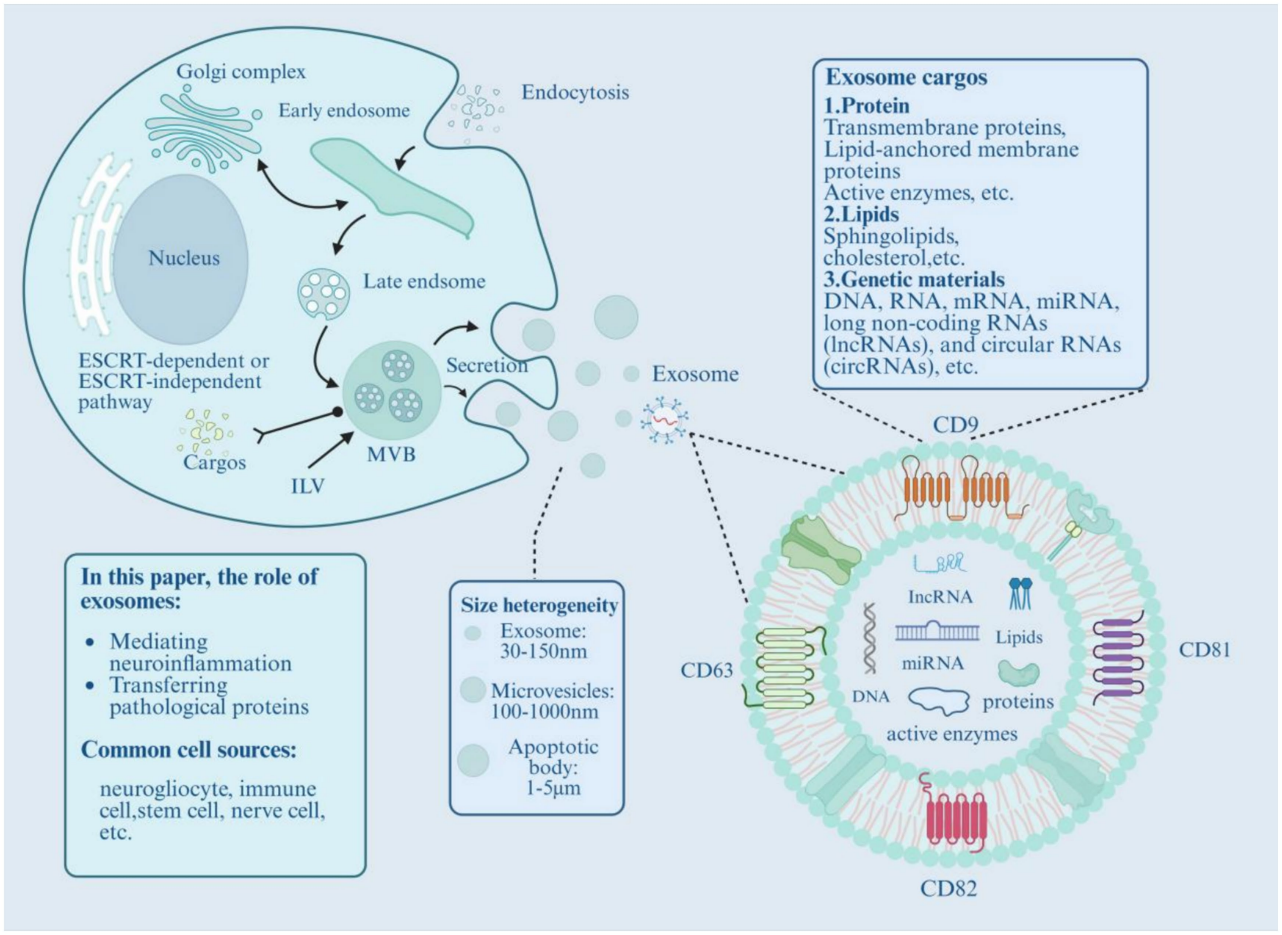
Schematic diagram of exosome biogenesis, cargo composition, and size heterogeneity. (1) Biogenesis pathway: The process starts with plasma membrane endocytosis (labeled “Endocytosis”) to form early endosomes (tubular structures in peripheral cytoplasm), which mature into late endosomes (spherical structures near the nucleus). Through ESCRT-dependent or independent pathways, the late endosomal membrane invaginates to generate intraluminal vesicles (ILVs, 40–150 nm), transforming into multivesicular bodies (MVBs). Finally, MVBs fuse with the plasma membrane (labeled “Secretion”) to release ILVs as exosomes. (2) Cargo composition: Exosomes encapsulate diverse bioactive molecules, including (1) Proteins (transmembrane proteins, lipid-anchored membrane proteins, active enzymes, etc.), (2) Lipids (sphingolipids, cholesterol, etc.), and (3) Genetic materials (DNA, RNA, mRNA, miRNA, lncRNA, circRNA, etc.). (3) Size comparison: Exosomes (30–150 nm) are smaller than microvesicles (MVs, 100–1,000 nm) and apoptotic bodies (1–5 μm). Membrane markers CD9, CD63, CD81, and CD82 (tetraspanin family) are labeled on the exosome surface. (4) Key symbols: MVB = multivesicular body; ILV = intraluminal vesicle; ESCRT = Endosomal Sorting Complexes Required for Transport; lncRNA = long non-coding RNA; circRNA = circular RNA. Common cell sources of exosomes include neuroglia, immune cells, stem cells, and neurons.

Exosomes are presently broadly defined as a class of EVs with physiological functional activity (35). Some vesicles are 30–150 nm in diameter (36, 37), while others, such as microvesicles (MVs), range from 100 to 1,000 nm (38, 39), with exosomes being categorized as a smaller subset of these vesicles. In addition, apoptotic bodies— the largest EVs— have diameters of approximately 1–5 μm (40). Beyond size differences, these three types of vesicles also differ subtly in their formation mechanisms, release pathways, composition, protein structure, and functions (35, 41). As a lipid bilayer membrane structure, exosomes can be secreted by almost all cell types (42, 43). Their biogenesis involves a multi-stage dynamic process based on endocytosis, centered on the generation and secretion of multivesicular bodies (MVBs) (see Figure 1) (44). The process begins with plasma membrane endocytosis: the cell membrane invaginates to form early endosomes, initially presenting as tubular structures distributed in the peripheral cytoplasm (45). As maturation progresses, early endosomes gradually convert into late endosomes characterized by structural reorganization (from tubular to spherical) and spatial repositioning (moving toward perinuclear regions) (42). During the late endosome maturation phase, membrane invagination is activated, triggering inward budding of the endosomal membrane via Endosomal Sorting Complexes Required for Transport(ESCRT)-dependent or independent pathways (46), producing intraluminal vesicles (ILVs) with diameters of 40–150 nm. At this stage, the late endosome transforms into a multivesicular body (MVB) containing multiple ILVs (47, 48). During this process, functional cellular components—including proteins, nucleic acids, and lipids—are selectively incorporated into ILVs, forming nanovesicles with specific molecular cargos (43). The final step involves the transport and secretion of MVBs: mature MVBs are transported along the cytoskeleton to the vicinity of the plasma membrane, where membrane fusion releases the ILVs into the extracellular space (see Figure 1) (49–51). Exosomes can be identified using transmission electron microscopy or by detecting specific marker proteins (51, 52). For instance, members of the tetraspanin family—such as CD9, CD63, and CD81—are highly enriched on the exosome membrane; they serve as classical markers for vesicles and also reflect the selective sorting processes during exosome formation (53). External stimuli—including cytokines, mechanical forces, and pathogen infections—also regulate exosome secretion by activating specific signaling pathways to enhance or inhibit the process (50). The formation and release of exosomes depend on multiple intracellular protein networks: Rab GTPases regulate exosome secretion, with Rab27a/b and phospholipase D1 (PLD1) precisely controlling MVB membrane fusion (54, 55). Studies show that depleting Rab27a reduces exosome secretion. The ESCRT complex is pivotal in cargo sorting, ILV formation, and MVB transport, with multiple protein complexes modulating inward budding (56). Disrupting ESCRT components affects exosome size, quantity, and cargo content (57). MVB targeting and transport involve small GTPases, associated protein complexes, motor proteins, and the cytoskeleton working together (58).

## Exosomal cargo

The composition of exosomal cargo is highly complex (37, 43). Studies have identified approximately 1,200 proteins in exosomes derived from various cells (53). According to the exocarta database (http://www.exocarta.org/), more than 10,000 exosome proteins have been collected from this database alone, in addition to more than 3,000 mrnas and lipids, respectively. These bioactive molecules reflect the cell of origin’s state (43, 48); for example, RNA sequencing reveals differences in tRNA species between exosomes from bone marrow and adipose tissue mesenchymal stem cells (MSCs), which relate to their source and differentiation status (59). Exosomes transport these molecules through the extracellular environment, where they can influence target cells and regulate biological activities such as growth, proliferation, differentiation, and apoptosis (58). For example, neurons expressing *α*-syn can release exosomes containing α-syn protein, which can transfer to other neurons, inducing abnormal protein aggregation in recipient cells (60). As natural carriers, exosomes harbor a wealth of bioactive substances within their lumen, including transmembrane proteins, lipid-anchored membrane proteins, and active enzymes (61). Lipids, including sphingolipids and cholesterol, are essential for distinguishing exosomes from lysosomes (45). Moreover, exosomes are precisely packed with genetic materials—including DNA, RNA, mRNA, miRNA, long non-coding RNAs (lncRNAs), and circular RNAs (circRNAs) (62). These highly heterogeneous molecular sets work collaboratively at multiple levels, forming a sophisticated molecular bridge for intercellular transfer of materials and signal transduction—among which miRNAs, cytokines, and inflammasome-related proteins are particularly critical in mediating the crosstalk between immune cells and neurons during neuroinflammation (see Figure 1) (38, 60, 63).

## Exosome-neuroinflammation crosstalk

### The non-inflammasome pathway

**Figure 2:**
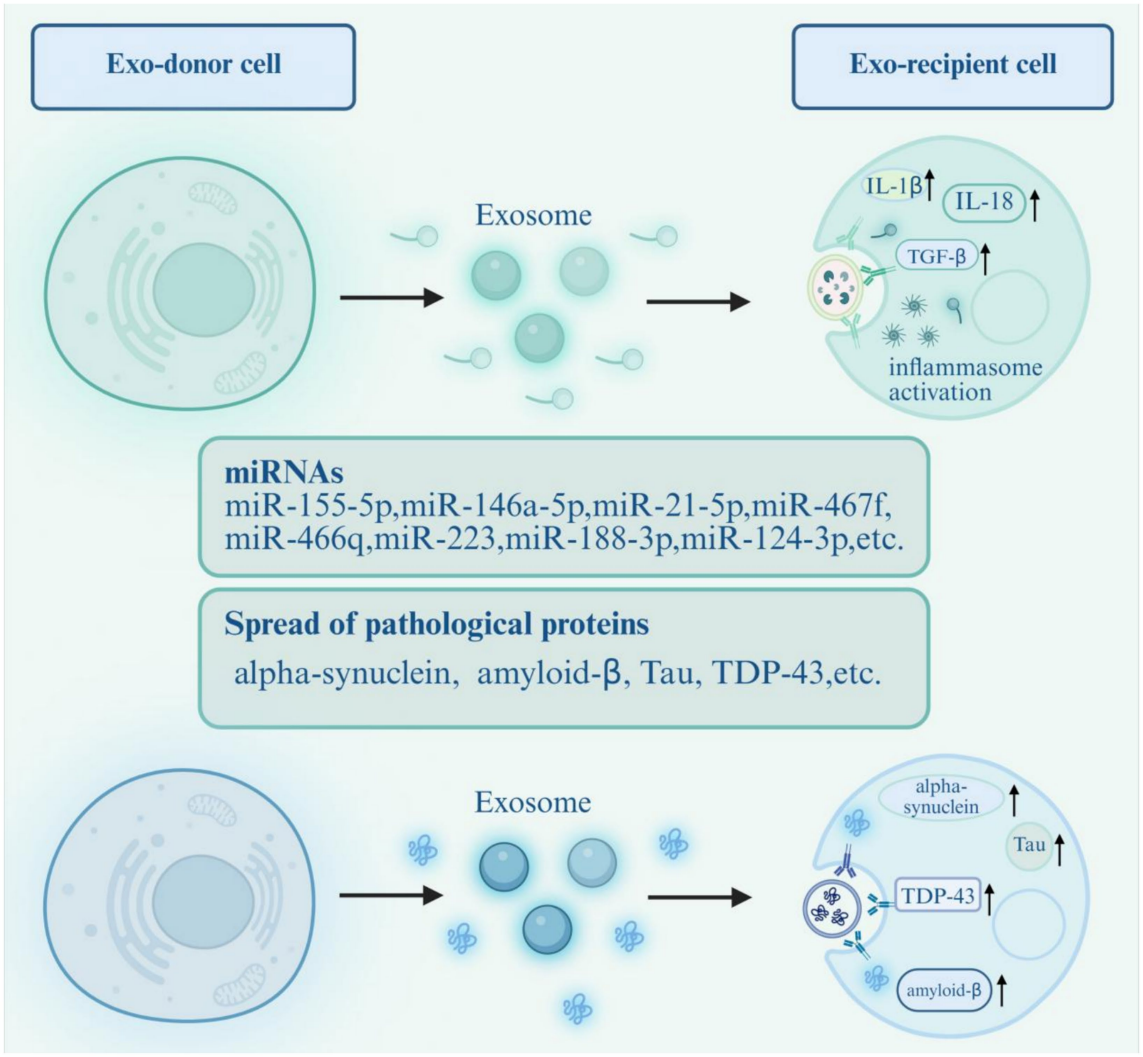
Dual roles of exosomes as mediators of neuroinflammation and pathological protein transmission. (1) Inflammatory driving mediators: Exosomes carry miRNAs (e.g., miR-155-5p, miR-146a-5p) or cytokines (e.g., IL-1β, IL-18, TGF-β) to regulate neuroinflammation. For example, miRNAs derived from inflammatory macrophages or microglia target signaling pathways (HIF1α/mtROS, TRAF6/NF-κB) to activate microglia/astrocytes or inhibit inflammasome activation. (2) Pathological protein transmission mediators: Exosomes encapsulate misfolded pathological proteins (α-synuclein [α-syn], amyloid-β [Aβ], Tau, TDP-43) and transport them to recipient neurons via membrane fusion or endocytosis, inducing abnormal aggregation and neuronal damage. Key symbols: miRNAs = microRNAs; IL = interleukin; TGF-β = transforming growth factor-β; NLRP3 = NOD-like receptor pyrin domain-containing 3; ASC = apoptosis-associated speck-like protein containing a CARD; α-syn = α-synuclein; Aβ = amyloid-β.

Exosomes can serve as mediators for intercellular transmission of inflammatory signals, exerting pro-inflammatory or anti-inflammatory effects through non-inflammasome-mediated cytokine secretion pathways. Among these, miRNAs are the primary cargo within exosomes that function as messengers in the interactions between immune cells and neurons (see Table 1; Figure 2) (64). Currently, some studies focus on the correlation between differential exosomal miRNA profiles and neuroinflammation. For example, in neurons derived from cells (iPSCs) differentiated from AD patients, the expression levels of miR-21 and miR-124 are significantly upregulated; similarly, these miRNAs are also enriched in exosomes. In exosomes derived from astrocytes, miR-21-5p expression is likewise increased, indicating enrichment of miR-21-5p in AD neuron- and astrocyte-derived exosomes. Conversely, miR-155 is upregulated in exosomes relative to its lower levels within cells, which may relate to susceptibility to neuroinflammation (65). Studies suggest that exosomes derived from inflammatory macrophages may contribute to the pathogenesis of PD (66). Exosomes extracted from RAW 264.7 cells treated with lipopolysaccharide (LPS) or interferon gamma (IFNγ), designated as LPS exosome (LPS-EXO) and IFNγ exosome (IFNγ-EXO), respectively, significantly increase the production of pro-inflammatory cytokines such as IL-1α, IL-1*β*, IL-2, IL-6, IL-12β, and TNFα in microglia. Similarly, LPS-EXO raises the expression of IL-1α, IL-1β, and IL-12α in astrocytes. These findings indicate that exosomes from inflammatory macrophages can induce microglia and astrocytes to produce pro-inflammatory cytokines, thereby promoting neuroinflammation (67). Additionally, five miRNAs—miR-155-5p, miR-132-3p, miR-146a, miR-210-3p, and miR-330-5p—have been identified in exosomes from inflammatory macrophages; these may play a vital role in PD pathogenesis by activating microglia and astrocytes to induce neuroinflammation and facilitate disease progression (67).

**Table 1:** Exosomes carry miRNAs to influence the progression of inflammation through related proteins or pathways.

Exosome cellular origin	miRNAs	Targeted proteins/pathways	Results	References
Infamed Macrophages	miR-155-5p	NA	The activation of microglia and astrocytes and the release of inflammatory factors	(67)
Microglial cell	NA	NLRP 3、ASC、caspase-1	Promote inflammasome activation and transmission	(78)
huc-MSCs/microglial cell	miR-146a-5p	miR-146a-5p/ TRAF6 axis, HIF1α/mtROS	Inhibit the activation of inflammasomes and microglial pyroptosis	(82, 79)
Neuron	miR-21-5p	iNOS and CD206	Microglia polarize to the M1 phenotype	(96, 65)
Mesenchymal stromal/stem cells (MSCs)	miR-467f, miR-466q	p38 MAPK	Inhibit the polarization of microglia into the M1 phenotype	(95)
microglial cell	miR-223	YB-1/miR-223 axis	The nerve damage was improved	(98)
Mesenchymal stromal/stem cells (MSCs)	miR-188-3p	CDK 5/NLRP3	Inhibit the activation of inflammasomes	(102)
hUC-MSCs	NA	NRF 2/NF-kB/NLRP 3	Inhibit the activation of inflammasomes	(100)
Microglial cell	miR-124-3p	mTOR signaling pathway and TLR4 signaling pathway	Modulating the M2 polarization of microglia	(97)

Other studies highlight that circulating exosomes act as mediators of neuroinflammation in NDDs (68, 69). For instance, exosomes isolated from the peripheral blood of aged C57BL/6 J wild-type mice, when injected via tail vein into healthy mice, are detected in various brain regions—particularly around blood vessels, cortex, and hippocampus—and lead to a significant increase in TGF-β mRNA levels 24 h post-injection. Correspondingly, the expression of glial activation-related genes such as Gfap and Cd68 also rises, suggesting that exosomes from aged mice may cross the BBB, influence TGF-β signaling pathways, and induce glial activation to mediate neuroinflammation (70). Furthermore, some studies demonstrate that using cell-permeable compounds that block exosome release can inhibit exosome synthesis or endocytosis in microglia, significantly reducing gene expression of TNFα and IL-1β induced by rotenone (71), further confirming the role of exosomes as mediators in neuroinflammation.

### Inflammasome pathway

Additionally, there is crosstalk between exosomes and inflammasomes (63). Pattern recognition receptors (PRRs), upon binding to exogenous pathogen-associated molecular patterns (PAMPs) or endogenous damage-associated molecular patterns (DAMPs), assemble into protein complexes known as “inflammasomes” (72). The recruitment of inflammasome components leads to the unconventional secretion of inflammatory cytokines such as IL-1β and IL-18. Studies have detected these cytokines within cell-derived exosomes, confirming that exosomes may serve as important unconventional secretion pathways and participate in inflammasome activation (73). Exosomes that promote or inhibit inflammasome activation often originate from various cell types; current studies and evidence mainly focus on immune cell-derived exosomes (74). Recent research has observed that microglia can efficiently secrete exosomes as mediators for antigen presentation and cargo release (75).

Long-term exposure to transition metals, such as manganese (Mn), iron, and copper—common in occupational or environmental settings— is associated with various NDDs, including AD and PD (76, 77), reported that exosomes released from microglia under Mn exposure contain large amounts of ASC (apoptosis-associated speck-like protein containing a CARD), a critical component of inflammasomes. These exosomes can activate the NLRP3 inflammasome and transfer this activation to other cells, thereby stimulating inflammatory responses in neighboring cells (78). Sarkar and colleagues found that Mn^2+^ ions can be a second signal to activate the NLRP3 inflammasome within microglia, leading to mitochondrial dysfunction and excessive mitochondrial superoxide production. Moreover, they reported that Mn^2+^ exposure enhances the exosomal release of ASC from LPS-primed microglia and significantly increases the number of exosomes released. The exosomal ASC content is higher compared to controls, and pro-inflammatory cytokines in the serum of workers exposed to welding fumes are elevated, supporting the role of exosomal ASC and IL-1*β* in neuroinflammation regulation (78).

In exosomes derived from microglia under intermittent hypoxia conditions, miR-146a-5p levels exhibit oscillating changes relative to hypoxia duration, suggesting a role in modulating neuroinflammation. *In vitro* studies demonstrated that miR-146a-5p suppresses NLRP3 inflammasome activation in neurons after intermittent hypoxia by targeting the HIF1*α*/mtROS signaling pathway (79). Additionally, EVs containing ASC isolated from serum of traumatic brain injury patients can upregulate inflammasome component expression and promote inflammasome activation in human pulmonary endothelial cells, further confirming that exosomes participate in modulating inflammasome activity (80).

Other research has shown that stem cell-derived exosomes can inhibit NLRP3 inflammasome-mediated inflammation and pyroptosis (81). For instance, miR-146a-5p is enriched in exosomes derived from human umbilical cord mesenchymal stem cells (huc-MSCs). These exosomes transfer miR-146a-5p to the spinal dorsal horn, leading to downregulation of TRAF6 (TNF receptor-associated factor 6) and reductions in NLRP3, ASC, cleaved caspase-1-p20, mature IL-1β, IL-18, and Gasdermin D (GSDMD)—all proteins involved in inflammation and microglial pyroptosis—indicating suppression of NLRP3 inflammasome activation and GSDMD-mediated pyroptosis (82, 83). The view of other studies is that inflammasomes are “enhancers” of exosome release, and inflammasomes or their activators can promote the secretion of exosomes; inflammasomes or their activators can promote extracellular vesicle secretion (63, 75). β-glucan, a significant component of fungal cell walls, induces a strong innate immune response and activates NLRP3 inflammasome assembly (84). Cypryk and colleagues employed proteomics to discover that, in human macrophages, β-glucan stimulation significantly enhances exosome release and exosome-mediated protein secretion. This process depends on Syk kinase activity, cysteine proteases within lysosomes, and caspase-1 activity, indicating that NLRP3 inflammasome activation also signals exosome secretion (85).

### Peripheral inflammation-CNS crosstalk mediated by exosomes

The crosstalk between peripheral inflammation and CNS pathology is a critical yet underexplored mechanism in NDD progression, with exosomes acting as key messengers that bridge the peripheral immune system and the CNS. Peripheral inflammatory stimuli (e.g., infection, environmental toxins, and metabolic disorders) induce immune cells to secrete exosomes carrying pro-inflammatory cargo, which can cross the BBB and trigger neuroinflammation (86). The underlying mechanisms involve three core steps: (1) Peripheral immune activation: Inflammatory triggers stimulate peripheral immune cells to upregulate pro-inflammatory cytokines (TNF-α, IL-1β) and package them into exosomes, along with miRNAs (miR-155-5p, miR-21) and DAMPs (71, 78, 87). (2) BBB penetration: Exosomes exploit multiple pathways to cross the BBB, including transcytosis via endothelial cells, receptor-mediated binding (e.g., CD9/CD63 interaction with BBB endothelial receptors), and uptake by perivascular microglia (88). (3) CNS inflammation initiation: Once in the brain, exosomal cargo activates resident microglia and astrocytes through TLR/NF-κB or NLRP3 inflammasome pathways, amplifying neuroinflammation and promoting pathological protein aggregation (89). This crosstalk has significant clinical translational implications. For example, in individuals exposed to environmental neurotoxins (e.g., Mn^2^ + in welding workers), peripheral inflammatory exosomes carrying ASC and miR-155-5p have been linked to increased PD risk, suggesting that targeting peripheral exosome secretion could serve as a preventive strategy (78).

### Exosomal miRNAs promote microglial polarization

During neuroinflammation, microglia serve as the primary immune cells of the central nervous system (CNS), playing essential roles (90). They clear cellular debris and pathogens and modulate inflammatory responses through cytokine and chemokine secretion. Exosomes may act as a missing link between chronic systemic inflammation and AD (91). Microglia respond to CNS injury and environmental changes with a complex “activation” (92). However, excessive microglial activation leads to overproduction of inflammatory mediators, resulting in neuronal damage and death. Activated microglia tend to polarize into two phenotypes: M1 and M2 (81). M1 microglia promote neuroinflammation, while M2 microglia suppress it. Modulating microglial polarization is a crucial mechanism in response to neuroinflammation (93). Although some studies argue that classifying microglia strictly into M1/M2 phenotypes does not fully capture their complexity, this classification helps clarify their neuroprotective versus neurotoxic roles (94).

Research indicates that exosomes can regulate microglial phenotypes— pro-inflammatory or anti-inflammatory — by transferring specific miRNAs (95). For example, exosomes derived from PC12 cells contain miR-21-5p. When microglia phagocytose these exosomes, they are induced to polarize towards the M1 phenotype, with increased expression of miR-21-5p within the microglia. This exacerbates the release of neuroinflammatory factors, accelerates neuronal injury, and promotes tau protein accumulation (96). Eight miRNAs in MSCs pretreated with IFN-*γ* are upregulated and detected in their derived exosomes. Transfecting specific miRNA mimics into microglia significantly impacts their inflammatory phenotype; for example, miR-467f and miR-466q inhibit the expression of their target genes MAP3K8 and MK2, respectively, suppressing the activation of the p38 MAPK pathway and thereby reducing microglial pro-inflammatory polarization (95). Furthermore, exosomes derived from microglia, particularly those containing miR-124-3p, play a vital role in regulating neuroinflammation and promoting neuroregeneration following traumatic brain injury (TBI) (97). These exosomes can suppress inflammatory M2 polarization and exert neuroprotective effects, improving neurological function in animal models. M2 microglia can release exosomes rich in miR-223, which can be taken up by AD cell models, leading to improvements in cell morphology, increased synaptic growth, and reduction of neuronal damage in AD models (98).

A large amount of stem cell-derived exosomes is also used to modulate microglial polarization and inhibit neuroinflammation to alleviate neurodegenerative disease progression (99). For example, exosomes derived from hUC-MSCs can reduce LPS-induced neuroinflammation by inhibiting the NRF2/NF-κB/NLRP3 signaling pathway (100). Experiments confirm that exosomes increase NRF2 levels and suppress LPS-induced phosphorylation of NF-κB p65 and NLRP3 inflammasome activation, promoting microglial polarization toward the M2 phenotype. The NRF2 inhibitor ML385 antagonizes the anti-inflammatory effects of exosomes (100). Exosomes derived from MSCs (MSC-Exo) can cross the BBB and be internalized by CNS parenchymal cells. MSC-Exo enhances M2 microglial polarization while reducing M1 polarization, thereby decreasing neuroinflammation. It significantly reduces the protein levels of TLR2, IRAK1, and phosphorylated NF-κB, inhibiting the TLR2 signaling pathway to further attenuate neuroinflammation (101). The study found that miR-188-3p exerts significant neuroprotective effects in MPTP-induced Parkinson’s disease mouse models and MPP + -induced MN9D cell models by inhibiting autophagy and pyroptosis through targeting CDK5 and NLRP3. miR-188-3p enriched in mesenchymal stem cell-derived exosomes not only significantly reduced damage to substantia nigra neurons but also enhanced cell proliferation and decreased apoptosis (102).

Beyond their role in regulating neuroinflammation, exosomes also serve as key mediators in another core pathological feature of NDDs: the intercellular spread of misfolded pathological proteins. This dual function links neuroinflammation and protein aggregation, two interconnected axes of disease progression.

### Exosomes promote the spread of pathological proteins

Misfolded proteins’ abnormal aggregation is a common pathological feature in various NDDs, such as the abnormal deposition of amyloid-*β* and tau proteins in AD, and the misfolding and aggregation of *α*-synuclein in PD (16, 103). The distribution and spread of these pathological proteins in the brains of affected individuals are not random but follow highly organized and predictable spatiotemporal patterns (104). For example, in PD, *α*-syn pathology is first identified in the olfactory bulb and the dorsal motor nuclei of the glossopharyngeal and vagus nerves. Subsequently, this pathology spreads from the brainstem to the midbrain and forebrain, eventually reaching the cerebral cortex (105). Current evidence suggests that exosomes can partially explain the transmission and dissemination of these pathogenic proteins. Recent studies have shown that abnormal protein aggregation in NDDs can propagate from one cell to another, with exosomes regarded as critical mediators (106). The spread of *α*-synuclein is thought to occur through multiple release and uptake mechanisms, with exosomes playing a key role in long-distance transmission of *α*-syn. On one hand, aggregated α-syn can be degraded via the autophagy-lysosome pathway within cells; more importantly, it can also be secreted into the extracellular environment through exosomes (107). Studies indicate that neurons overexpressing *α*-syn can release exosomes carrying α-syn, transferring the protein to other healthy neurons, leading to aggregation and inducing target cell death(see Figure 2) (108). Ishiguro and colleagues found that levels of fibrillar α-syn are significantly increased in EVs from the serum of PD patients. When co-cultured with human α-syn preformed fibrils (PFFs), H4 cells produce EVs containing PFFs (PFFs-Exo), which have been shown through *in vitro* and *in vivo* experiments to promote rapid uptake and spreading of α-syn PFFs. PFFs-Exo induce increased phosphorylated α-syn-positive aggregates in the mouse brain and can transfer α-syn PFFs from the periphery to the central nervous system via intravenous injection (109). In AD, exosomes containing oligomeric Aβ isolated from AD brains are taken up by SH-SY5Y cells, resulting in cytotoxicity and delivery to other recipient cells (110). Microglia may also contribute to tau diffusion between neurons by uptake and release of exosomes containing pathological forms of Tau (111, 112).

### Exosomes mediate neuroinflammatory-pathological protein interactions

Previous studies have highlighted the role of amyloid-β, Tau, and α-syn aggregates in the pathogenesis, prognosis, and treatment of NDDs by triggering neuronal inflammation by activating several inflammation-related signaling pathways (113). For example, it has been found that NLRP3 inflammasome is significantly co-localized with neurofibrillary tangles and is significantly increased in AD (114). Tau monomers or oligomers, Aβ fibrils or oligomers can also activate microglia by activation of NLRP3 (115). Recent studies have shown that induced peripheral inflammation enhances *α*-Syn oligomer-mediated central neuroinflammation (116). However, the “mediator” of the interaction between pathological protein aggregation and neuroinflammation is still unknown. Recent studies have explored the relationship between exosomes and pathological protein aggregation as an intermediate link (113).

Harischandra and colleagues found that Mn^2+^ accelerates the intercellular exosomal delivery of α-syn, resulting in Mn^2+^-exposed DA neurotoxicity in the α-syn-A53T transgenic rat model. The team also found that in DA neurons expressing human α-syn, misfolded α-syn was secreted into extracellular mediators via exosomes after exposure to Mn^2+^ (117). These exosomes are endocytosed by microglia, thereby exacerbating the neuroinflammatory response (101). Microglia-derived exosomes also play an active role in the spread of *α*-syn. Both exogenous α-syn, introduced externally, and endogenous α-syn, released from neurons, can provoke strong neuroinflammatory responses from microglia, leading to the secretion of pro-inflammatory cytokines. The propagation of α-syn can be enhanced by inflammatory factors (31). Guo and colleagues co-cultured neurons and microglia with exosomes under both the presence and absence of inflammatory cytokines such as TNF-α, IL-1β, and IL-6. Results showed that insoluble endogenous α-syn increased within neurons, and this effect was more pronounced under inflammatory conditions, indicating that TNF-α, IL-1β, and IL-6 can promote intracellular α-syn deposition, with microglial inflammatory responses accelerating this process. Inhibition of microglial exosome production with GW4869 significantly reduced the release of microglia-derived exosomes containing α-syn and decreased its propagation in neurons. Depleting microglia using PLX3397 markedly suppressed α-syn transmission and neurodegeneration (31). Other studies have shown that exosomes can carry CryAB^R120G^ protein through the periphery to the brain and trigger prion-like transmission behavior in the brain, which leads to abnormal accumulation of CryAB protein in the brain and activation of NLRP3 or NLRP3-ASC inflammasome in glial cells and exacerbates neuronal damage (118).

### Exosomes as therapeutic vectors

Leveraging their inherent biological properties, exosomes have emerged as promising therapeutic vectors for NDDs, with broad application prospects in drug delivery and gene therapy (119). Their key advantages include natural BBB penetration, low immunogenicity, and high biocompatibility, which address the major bottlenecks of traditional therapeutic agents (120).

In drug delivery, exosomes can be engineered to encapsulate various therapeutic agents, including small-molecule drugs, peptides, and proteins. For example, exosomes loaded with curcumin (a natural anti-inflammatory and antioxidant compound) have been shown to reduce Aβ aggregation and neuroinflammation in AD mouse models by targeting microglia (121). Similarly, exosomes carrying levodopa exhibit prolonged circulation time and enhanced brain accumulation compared to free levodopa, improving therapeutic efficacy while reducing systemic side effects (122).

In gene therapy, exosomes serve as safe carriers for nucleic acid-based therapeutics such as miRNAs, siRNAs, and lncRNAs. For instance, MSC-derived exosomes overexpressing miR-124 have been demonstrated to suppress microglial M1 polarization and promote neuronal repair in ALS models by targeting TLR4 signaling (123). Additionally, exosomes delivering siRNA against *α*-syn have successfully reduced pathological α-syn aggregation in PD mice, highlighting their potential to interrupt the prion-like spread of misfolded proteins (124). Clinical translation of exosome-based therapies is progressing steadily. Several preclinical studies have validated the feasibility of exosome-mediated delivery in large animal models, and early-phase clinical trials are investigating the safety and efficacy of MSC-derived exosomes (125). However, challenges remain, including standardized large-scale production, targeted modification efficiency, and long-term safety evaluation, which require further optimization for clinical application.

## Conclusion

Over the past decade, exosomes have emerged as critical mediators in the pathogenesis of NDDs, bridging the gap between neuroinflammation and pathological protein propagation. These nanovesicles serve dual roles: as “pathological shuttles” facilitating the intercellular transmission of toxic proteins (e.g., Aβ, Tau, α-syn) and as dynamic regulators of neuroinflammatory cascades. By shuttling misfolded proteins across cells, exosomes enable the spatiotemporal spread of pathology observed in AD, PD, and related disorders. Simultaneously, exosomal cargo—including miRNAs, cytokines, and inflammasome components—modulates microglial polarization, NLRP3 inflammasome activation, and cytokine release, creating a vicious cycle that amplifies neuronal damage. For instance, exosomal miR-21-5p and miR-146a-5p regulate neuroinflammatory responses by targeting pathways like HIF1α/mtROS or TRAF6, while ASC-containing exosomes propagate inflammasome activation across cells. Conversely, stem cell-derived exosomes exhibit therapeutic potential by promoting anti-inflammatory M2 microglial polarization via miR-223 or NRF2/NF-κB signaling, highlighting their bidirectional regulatory capacity.

### Future perspectives

Despite significant progress in unraveling exosome-mediated mechanisms in NDDs, several critical and challenging scientific questions and technical bottlenecks remain to be addressed. First, regarding exosomal heterogeneity—one of the most intractable barriers—current isolation techniques fail to distinguish functionally distinct exosome subpopulations. Technical bottlenecks include the lack of high-throughput single-exosome analysis tools capable of simultaneously profiling surface markers, cargo composition, and biological activity, which hinders the identification of disease-specific “pathological exosome signatures” with diagnostic or prognostic value. Furthermore, the molecular mechanisms underlying cargo sorting specificity remain elusive, requiring integration of single-cell sequencing and super-resolution microscopy to dissect cell-type-specific biogenesis pathways.

Second, translating preclinical findings to human NDDs faces substantial hurdles. Animal models often recapitulate only partial pathological features, and exosome-mediated protein transmission observed *in vitro* lacks direct validation in human longitudinal studies. A key scientific question is: Do circulating exosomal cargoes (e.g., miR-155-5p, fibrillar α-syn) serve as causal mediators or merely correlative biomarkers of disease progression? Addressing this requires large-scale prospective cohorts with standardized sample processing and multi-omics integration to validate exosomal signatures that robustly distinguish disease subtypes, stages, and treatment responses. Additionally, the high variability of human clinical samples complicates the identification of consistent exosomal biomarkers, demanding advanced statistical models to control for confounding factors.

Third, the molecular mechanisms underlying exosome-mediated crosstalk between peripheral inflammation and CNS pathology remain poorly defined and represent a critical knowledge gap. Key unanswered questions include: What specific BBB endothelial receptors mediate exosome transcytosis? How do environmental triggers regulate exosome secretion from peripheral immune cells and alter their cargo sorting to enhance neuroinflammatory potential? Furthermore, the bidirectional crosstalk between exosomal pathological proteins and neuroinflammation—whether exosomal α-syn induces inflammation first or inflammatory cues promote exosomal protein packaging.
